# Development of a Transformation System and Locus Identification Pipeline for T-DNA in *Chrysanthemum seticuspe*, A Model Species for Hexaploid Cultivated Chrysanthemum

**DOI:** 10.3390/ijms231911426

**Published:** 2022-09-28

**Authors:** Jiali Zhang, Jing Zhang, Peiling Li, Yuan Gao, Qi Yu, Daojin Sun, Lingling Zhang, Siqi Wang, Jing Tian, Zhenxing Wang, Jiafu Jiang, Fadi Chen, Aiping Song

**Affiliations:** 1 State Key Laboratory of Crop Genetics and Germplasm Enhancement, Key Laboratory of Landscaping, Key Laboratory of Flower Biology and Germplasm Innovation (South), Ministry of Agriculture and Rural Affairs, Key Laboratory of Biology of Ornamental Plants in East China, National Forestry and Grassland Administration, College of Horticulture, Nanjing Agricultural University, Nanjing 210095, China; 2 Henan Key Laboratory of Tea Comprehensive Utilization in South Henan, Xinyang Agriculture and Forestry University, Xinyang 464000, China; 3 Central Laboratory of College of Horticulture, Nanjing Agricultural University, Nanjing 210095, China

**Keywords:** *Agrobacterium* *tumefaciens*, antibiotics, *Chrysanthemum seticuspe* Gojo-0, in vitro regeneration, identification of insertion locus

## Abstract

Chrysanthemum is one of the most popular flowers worldwide and has high aesthetic and commercial value. However, the cultivated varieties of chrysanthemum are hexaploid and highly heterozygous, which makes gene editing and gene function research difficult. Gojo-0 is a diploid homozygous line bred from a self-compatible mutant of *Chrysanthemum seticuspe* and is expected to become a model plant of the genus *Chrysanthemum*. After assessment of different growth regulator combinations, the optimal concentrations of α-naphthaleneacetic acid (NAA) and 6-benzyladenine (6-BA) in the regeneration system were 1.0 mg·L^−1^ and 0.2 mg·L^−1^, respectively. In the genetic transformation system, the selected concentrations of kanamycin, hygromycin and glufosinate-ammonium were 10 mg·L^−1^, 2.5 mg·L^−^^1^ and 0.6 mg·L^−1^ for bud generation and 12 mg L^−1^, 1.5 mg·L^−1^ and 0.5 mg·L^−1^ for rooting. The transgenic plants were verified by not only PCR detection and GUS staining, but also identification of the T-DNA insertion locus using high-throughput sequencing. Our results lay the foundation for gene functional research on chrysanthemum and will help with the identification of transgenic plants.

## 1. Introduction

Chrysanthemum is a famous traditional flower in China; it has several varieties, gorgeous flower colors and high ornamental and application value. It is also one of the most commercially valuable flower crops in the global flower industry. However, due to the self-incompatibility of cultivated chrysanthemum, the genome is highly heterozygous. Most cultivated chrysanthemum varieties exhibit complex hexaploidy, which leads to difficulties in genetic analysis. Cultivated chrysanthemum varieties are often formed by natural hybridization of wild chrysanthemum species and artificial breeding. Wild *Chrysanthemum* species usually have similar flower morphologies and growth and flowering characteristics, especially *Chrysanthemum seticuspe,* which has been used to analyze flowering regulation in chrysanthemum [1,2].

Nakano et al. bred a self-compatible mutant plant from *C. seticuspe* through multiple generations of self-breeding and selection, establishing a pure lineage named Gojo-0. Gojo-0 not only has the excellent characteristics of wild chrysanthemum, but also has the characteristics of self-compatibility and genomic homozygosity [3]. Self-compatibility leads to homozygous recessive mutations, and mutant phenotype expression and genome purity eliminate the allelic redundancy. All of these factors make Gojo-0-based genetic analysis simple compared with that for hexaploid chrysanthemums, which facilitates mutant formation, the separation of natural mutants and genetic analysis. Gojo-0 was developed as a model strain for molecular research on *Chrysanthemum* species. The publication of the Gojo-0 genome is of great significance for gene-editing research. The chromosome-level whole-genome sequence and plant resources of Gojo-0 can contribute to the analysis of interesting traits in the family Asteraceae and genus *Chrysanthemum* and facilitate modern breeding programs based on marker-assisted selection (MAS) and genome editing [4]. Hirakawa et al. performed linkage map construction, gene discovery and comparative analyses for *C.* *seticuspe* and cultivated chrysanthemum [5]. Single-nucleotide polymorphism identification and annotation of the *C. morifolium* genome showed that the *C. seticuspe* genome could be used for the genetic analysis of cultivated chrysanthemum. Therefore, molecular genetic research based on Gojo-0 will contribute to chrysanthemum breeding.

The application of traditional breeding methods in chrysanthemum is limited by many factors, such as long breeding periods, low seed yield, less favorable variations, and a lack of effective directional breeding tools. The development of genetic engineering has made genetic modification an important direction for the breeding of new varieties, making it possible to analyze the detailed functions of foreign genes of interest [6]. Among them, the *Agrobacterium*-mediated method using leaf discs as explants is the most widely used. To date, the *Agrobacterium*-mediated transformation system has been established in different chrysanthemum varieties [7]. Nakano et al. reported the generation of transgenic lines of Gojo-0, but the detailed genetic transformation process has not been described [3]. However, the low efficiency of genetic transformation limits the acquisition of transgenic *C. seticuspe*, so it is very important to construct an appropriate genetic transformation system [8]. In this study, we explored the effects of media containing growth hormones at different ratios on the regeneration of *C. seticuspe*, examined the effects of different antibiotics and selection pressures on bud regeneration and rooting, and standardized the genetic transformation system of Gojo-0. The *Agrobacterium*-mediated method was used to genetically transform Gojo-0 to obtain kanamycin-resistant transgenic plants with β-glucuronidase activity. This study will establish a better foundation for further research on the molecular regulatory mechanism and breeding of chrysanthemum.

## 2. Results and Discussion

To date, some regeneration and genetic transformation systems of chrysanthemum have been reported [9,10,11,12,13]. However, the most important factors affecting the success of *Agrobacterium*-mediated transformation are the characteristics of the varieties, and the factors affecting the transformation efficiency vary among varieties and species [14,15,16,17]. The explant type and age of chrysanthemum are the key factors affecting in vitro plant regeneration, and the use of young explants, such as newly expanded leaves, is a good material for transgenic chrysanthemum [18,19]. Based on previous reports, in this study, we selected hormones, the susceptible *Agrobacterium* strain EHA105 [20,21] and antibiotic types [10] suitable for the in vitro regeneration of chrysanthemum, and we developed a simple and effective leaf explant regeneration and genetic transformation system. To improve the transformation efficiency of Gojo-0, we optimized the system by analyzing several common factors affecting the transformation efficiency.

### 2.1. Role of Various Growth Regulators in Shoot Regeneration

**Figure 1:**
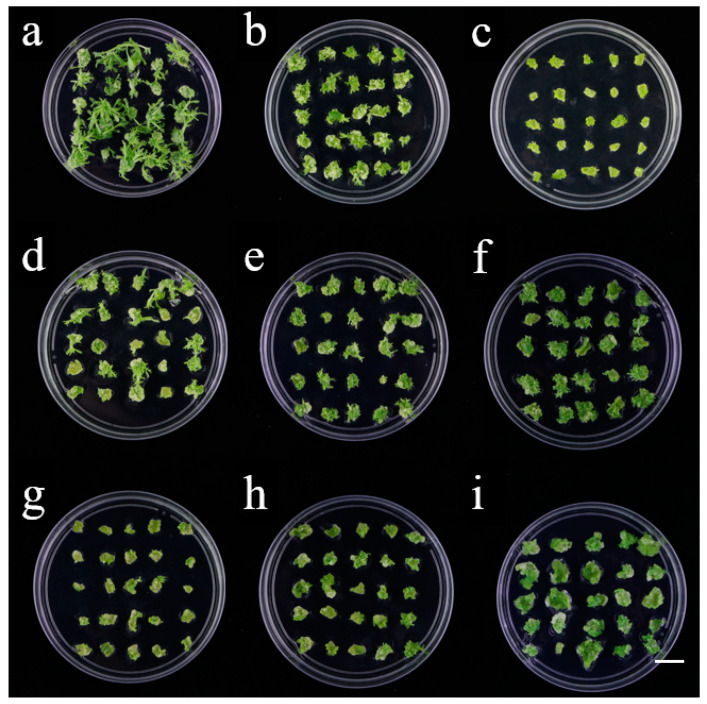
Effects of different hormone proportions on the regeneration of Gojo-0 (Bar = 2 cm). (**a**): M1 (6-BA:NAA = 1:0.2); (**b**): M2 (6-BA:NAA = 1:0.5); (**c**): M3 (6-BA:NAA = 1:0.8); (**d**): M4 (6-BA:NAA = 2:0.2); (**e**): M5 (6-BA:NAA = 2:0.5); (**f**): M6 (6-BA:NAA = 2:0.8); (**g**): M7 (6-BA:NAA = 3:0.2); (**h**): M8 (6-BA:NAA = 3:0.5); (**i**): M9 (6-BA:NAA = 3:0.8).

**Figure 2:**
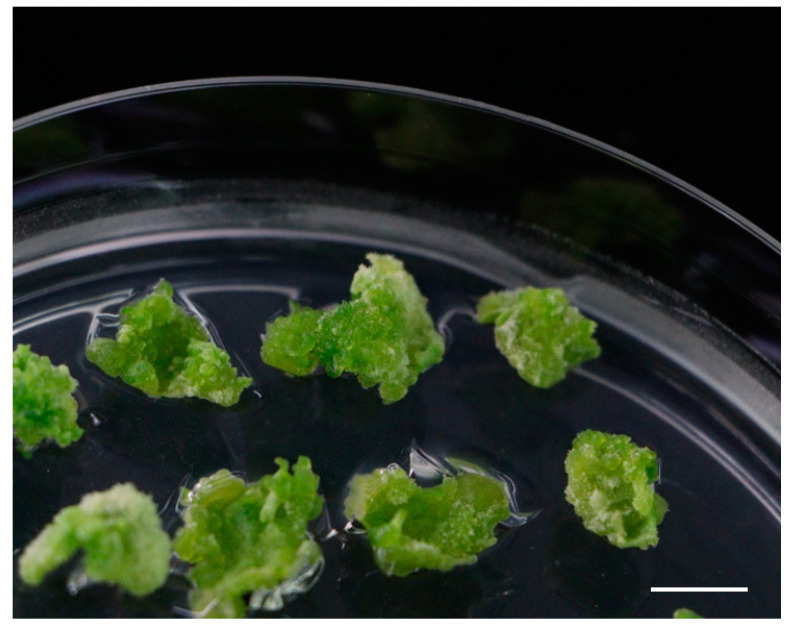
Leaf disc explants of Gojo-0 appeared vitrified on M9 medium (Bar = 1 cm).

In plant tissue culture and development, plant growth regulators have a great influence on the formation of adventitious buds in plant explants [22,23,24]. Therefore, the type and concentration of plant growth regulators are often key to optimizing transformation systems [25,26,27]. By screening for shoot regeneration under nine different combinations of 6-BA and NAA at different concentrations, as shown in the figure and table, we found that Gojo-0 could differentiate into buds on shoot regeneration medium with different concentration ratios of hormones, but there were great differences in the differentiation effects between different regeneration media (Table 1 and Figure 1). Among them, the induction effect of Gojo-0 treated with M1 (MS + 1.0 mg·L^−1^ 6-BA + 0.2 mg·L^−1^ NAA) was the best. Figure 1a shows that the largest volume of callus differentiation occurred in M1. The bud regeneration rate of leaf discs was the highest, and the length of buds was significantly longer than that of other leaf discs in the same period, reaching 220%, which was significantly higher than that with other growth regulator concentrations. Gojo-0 performed worst on M7, with showing the lowest amount of callus differentiation and yellow–green staining. Vitrification occurred on the M8 and M9 media, especially on the M9 medium (Figure 2).

As previously shown in some studies, Li et al. reported that a concentration ratio of 2:1 of cytokinin to auxin was conducive to the formation and differentiation of chrysanthemum leaf explants, while a high concentration of 6-BA (5 mg·L^−1^) and a low concentration of NAA (0.1 mg·L^−1^) were conducive to the induction and differentiation of shoot tip thin-cell-layer explants [28]. Similarly, Wang et al. reported the highest germination rate of ground-cover chrysanthemum on MS medium containing 2.0 mg·L^−1^ 6-BA and 0.5 mg·L^−1^ NAA [29]. Li et al. showed that the most efficient regeneration medium for leaf explants of eight chrysanthemum cultivars was MS + 6-BA 1.0 mg L^−1^ + NAA 0.5 mg L^−1^ [8]. In this study, our results were different from others, and we showed that the most efficient regeneration medium for Gojo-0 was MS + 1.0 mg·L^−1^ 6-BA + 0.2 mg·L^−1^ NAA.

### 2.2. Effect of Various Antibiotics on Shoot Regeneration and Rooting

**Figure 3:**
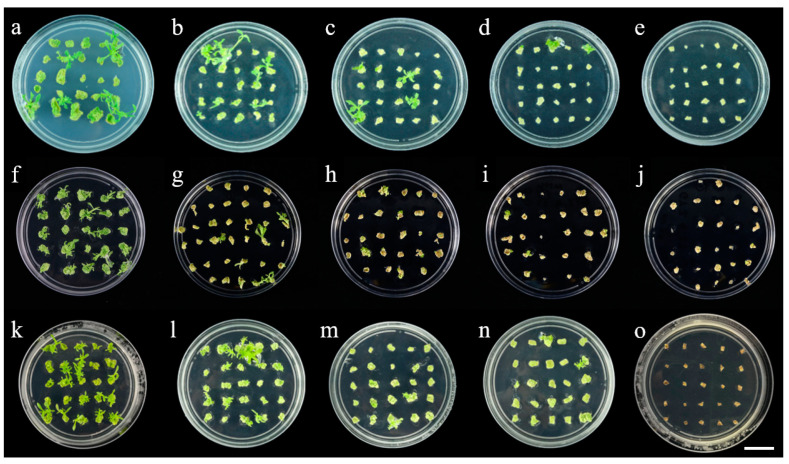
Effects of different concentrations of antibiotics on leaf disc explant differentiation of Gojo-0 (Bar = 2 cm). (**a**) Kanamycin 0 mg·L^−1^; (**b**) kanamycin 4.0 mg·L^−1^; (**c**) kanamycin 6.0 mg·L^−1^; (**d**) kanamycin 8.0 mg·L^−1^; (**e**) kanamycin 10.0 mg·L^−1^; (**f**) hygromycin 0 mg·L^−1^; (**g**) hygromycin 1.0 mg·L^−1^; (**h**) hygromycin 1.5 mg·L^−1^; (**i**) hygromycin 2.0 mg·L^−1^; (**j**) hygromycin 2.5 mg·L^−1^; (**k**) glufosinate-ammonium 0 mg·L^−1^; (**l**) glufosinate-ammonium 0.3 mg·L^−1^; (**m**) glufosinate-ammonium 0.4 mg·L^−1^; (**n**) glufosinate-ammonium 0.5 mg·L^−1^; (**o**) glufosinate-ammonium 0.6 mg·L^−1^.

**Figure 4:**
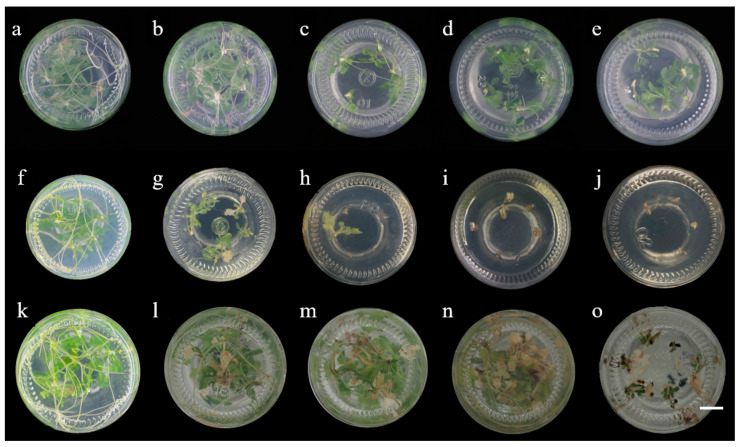
Effects of different concentrations of antibiotics on shoot rooting of Gojo-0 (Bar = 1 cm). (**a**) kanamycin 0 mg·L^−^**^1^**; (**b**) kanamycin 6.0 mg·L^−1^; (**c**) kanamycin 8.0 mg·L^−1^; (**d**) kanamycin 10.0 mg·L^−1^; (**e**) kanamycin 12.0 mg·L^−1^; (**f**) hygromycin 0 mg·L^−1^; (**g**) hygromycin 1.5 mg·L^−1^; (**h**) hygromycin 2.0 mg·L^−1^; (**i**) hygromycin 2.5 mg·L^−1^; (**j**) hygromycin 3.0 mg·L^−1^; (**k**) glufosinate-ammonium 0 mg·L^−1^; (**l**) glufosinate-ammonium 0.2 mg·L^−1^; (**m**) glufosinate-ammonium 0.3 mg·L^−1^; (**n**) glufosinate-ammonium 0.4 mg·L^−1^; (**o**) glufosinate-ammonium 0.5 mg·L^−1^.

According to the various transformation vectors used, we selected three antibiotics, namely, kanamycin, hygromycin and glufosinate-ammonium, all of which have inhibitory effects on the leaf disc regeneration of Gojo-0. With the increase in the antibiotic concentration, the leaf disc gradually turned white and then brown, and the callus differentiation rate decreased. This indicates that antibiotics inhibit the formation of new cells or calli until the explant dies [30]. Therefore, the selection of appropriate antibiotics and concentrations plays an important role in shoot regeneration and rooting [31,32]. Table 1 and Figure 3 shows that when the concentration of kanamycin reached 10 mg·L^−1^, the concentration of hygromycin reached 2.5 mg·L^−1^, the concentration of glufosinate-ammonium reached 0.6 mg·L^−1^, and the germination rate was 0. Similarly, kanamycin, hygromycin and glufosinate-ammonium also have a significant inhibitory effect on the rooting of Gojo-0 buds (Table 2 and Figure 4). With the increase in hygromycin and glufosinate-ammonium concentrations, the rooting of plant shoots was inhibited, or the shoots became white and died; however, kanamycin only prevented the rooting of Gojo-0 and did not cause albinism. The rooting of the shoots of Gojo-0 was completely inhibited when the concentrations of kanamycin, hygromycin and glufosinate-ammonium reached 12 mg·L^−1^, 1.5 mg·L^−1^ and 0.5 mg·L^−1^, respectively. Therefore, these concentrations of antibiotics were the critical tolerance values for leaf disc differentiation and bud rooting, and the transformation efficiency at this time was between 4 and 6%. Young Gojo-0 sterile seedlings aged approximately 40 days were cut into leaf discs, which can be used as a suitable selection in the genetic transformation of Gojo-0 in the future.

Among the three antibiotics used for screening, hygromycin had the highest toxicity toward Gojo-0, as evidenced by the death of the explants of Gojo-0 under a low concentration of hygromycin. The time to death was the shortest for hygromycin, followed by glufosinate-ammonium, while kanamycin needed a relatively high time and concentration. This is because hygromycin inhibits the binding of 80 S ribosomal subunits, leading to translation errors, and the number of subunits is high. Binding to hygromycin hinders the growth of explants. An increased amount of protein leads to the stagnation of metabolic processes and the death of explants [30]. Compared with other research results, it was reported that 15–25 mg·L^−1^ kanamycin completely inhibited callus induction and led to explant browning [28]. In this study, 10 mg·L^−1^ kanamycin was the optimal concentration for screening. He et al. reported that leaf disc explants of the chrysanthemum variety ‘Jinba’ were sensitive to hygromycin. They also found that the optimal concentration of hygromycin for callus selection was 8 to 10 mg·L^−1^ and that the optimal concentration for rooting selection was 18 mg·L^−1^ [33]. Kumar et al. reported that 30 mg L^−1^ kanamycin was best for the selection of *C. morifolium* var. Vivid Scarlet plants [34]. However, it was found that Gojo-0 was very sensitive to hygromycin in our preliminary experiment, and a low concentration of hygromycin inhibited explant healing and differentiation. This finding indicates that different chrysanthemum plants have different sensitivities to hygromycin. To date, there have been few reports on the application of glufosinate-ammonium as an antibiotic in chrysanthemum genetic studies. This study explored the effects of different concentrations of glufosinate-ammonium on the healing and differentiation of chrysanthemum leaf disc explants. Considering the low cost of glufosinate-ammonium, the results showed that 0.5–0.6 mg·L^−1^ glufosinate-ammonium was the optimal concentration, which provided a valuable reference for optimizing the genetic transformation system of chrysanthemum.

In addition, the interval between medium changes during the selection period was 2 weeks, and the medium could not provide sufficient nutrients beyond two weeks, so the plant tissue could no longer grow.

### 2.3. Molecular and Histochemical Analyses of Transgenic Plants

**Figure 5:**
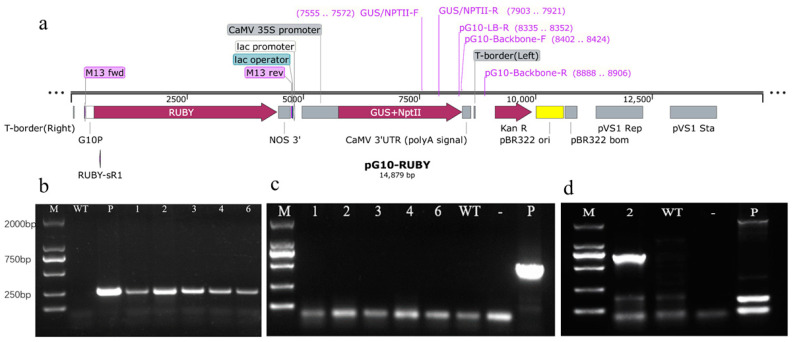
(**a**) pG10 vector map; (**b**) PCR analysis of transgenic plants of Gojo-0; (**c**) evaluation of the pG10 vector backbone by agarose gel electrophoresis; (**d**) evaluation of the insertion site of line 2 by agarose gel electrophoresis. M, DNA Marker DL 2000; WT, wild-type plant; -, negative control; P, PG10-GUS plasmid (positive control); 1–6, transgenic plants.

**Figure 6:**
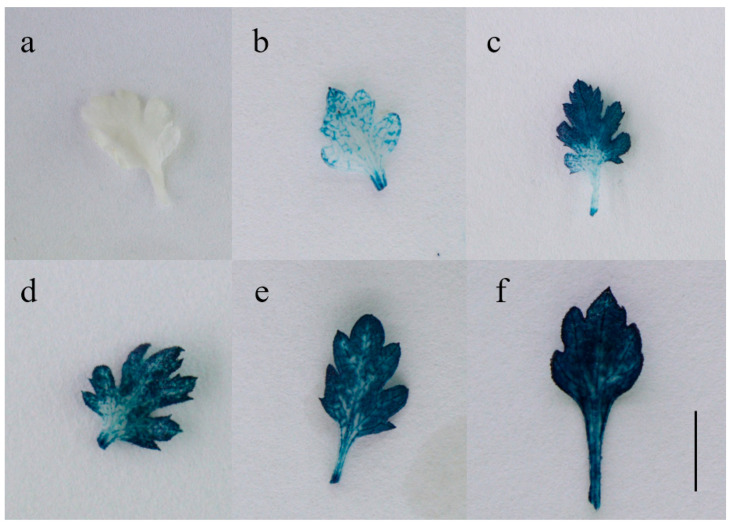
Histochemical evaluation of GUS expression in leaves of transgenic plants. (**a**) Control; (**b**–**f**) transgenic plants (Bar = 1 cm).

One T0 plant with good growth potential was selected from each rooting line and examined by PCR. The DNA of the untransformed plant was the negative control, and the plasmid DNA of *Agrobacterium* was the positive control. As seen from the Figure 5b, the amplified fragment size from five resistant seedlings was the same as that from the plasmid, and there was no amplification band in the negative control. Primers were designed on the vector backbone sequence for PCR amplification to verify whether the above bands were caused by bacterial residues (Figure 5a ). The results showed that only the plasmid amplified the band, and there was no band in the transgenic lines or the negative control (Figure 5c), which indicated that the target gene was successfully transferred into Gojo-0. Leaves of the PG-10 transgenic PCR-positive strain were selected for GUS staining, and leaf staining was observed under a microscope (Figure 6). After several staining observations, it was found that the leaves of the five transgenic lines were stained blue, which showed that the expression of GUS reached the level needed for staining. The results of this study showed that the *Agrobacterium*-mediated genetic transformation system of Gojo-0 with leaves as the transformation receptor was successfully constructed.

### 2.4. T-DNA Insertion Locus of pG10-2

We obtained 36.0 Gb PE150 Illumina reads, covering an approximately 12-fold genomic sequence of *C. seticuspe*. The detailed sequencing data are listed below. The raw read number was 240,036,600, the raw base number was 36,005,490,000, the clean read number was 238,581,710, the clean base number was 35,787,256,500, the raw Q30 base rate was 87.97%, and the clean Q30 base rate was 87.95%. All the assembled contigs were uploaded in the Supplemental Files, and the longest contig was 9058 bp, which was longer than the left border (LB) to right border (RB) region of the pG10 plasmid. After sequence alignment, we obtained a 269 bp flanking sequence from the LB. Finally, we found that the T-DNA was inserted into Cse2.0_LG3: 24,085,670 in the *C. seticuspe* genome. The result confirmed that the T-DNA was inserted into the genome. Then, we verified the results of high-throughput sequencing by PCR amplification (Figure 5d). Moreover, compared to the previous protocol for transformation [10], our detailed protocol provides a reference for the identification of insertion sites in other transgenic plants.

## 3. Materials and Methods

### 3.1. Materials

Gojo-0 (*C. seticuspe*) seeds were obtained from the Graduate School of Integrated Sciences for Life, Hiroshima University, 1-4-3, Kagamiyama, Higashi-Hiroshima, Japan [3]. After disinfection and sterilization with 75% (*v*/*v*) ethyl alcohol, the sterilized seeds were germinated in Murashige and Skoog (MS) medium [35]. When the seedlings grew, their shoots were removed and cultured in MS medium containing 3.0% sucrose and 0.32% plant gel that was adjusted to pH 5.8 before autoclaving at 116 °C for 30 min; samples were then incubated under a cold white fluorescent lamp (80–100 μmol·m^−2^·s^−1^) with a 16 h light cycle at 23 °C [3,28]. The bacterial strain *Agrobacterium tumefaciens* EHA105 harboring the pG10 plasmid was obtained from the State Key Laboratory of Crop Genetics and Germplasm Enhancement, Nanjing Agricultural University [36]. The MS media used for tissue culture experiments were provided by Hopebio (Qingdao, China). The plant gel used for tissue culture experiments was provided by Dingguo Biotech Co. (Beijing, China). In the process of transgene creation, agar was used to replace the plant gel at 0.7% (*w*/*v*). The hygromycin used in the experiment was obtained from Solarbio (Beijing, China), glufosinate-ammonium was obtained from Bayer (Leverkusen, Germany), and other plant hormones and antibiotics were obtained from Huayueyang Biotech Co. (Beijing, China).

### 3.2. Assessment of Growth Regulators of the Untransformed Plants

Improving the regeneration ability of chrysanthemum is the basis for improving the efficiency of genetic transformation. In this experiment, we explored the effect of the combination of 6-benzyladenine (6-BA) and α-naphthaleneacetic acid (NAA) on adventitious bud regeneration. The Gojo-0 regeneration system was screened using 9 regenerative MS media (Table 3) with different concentration ratios of 6-BA and NAA. The young leaves of 35- to 45-day-old sterile seedlings were selected as explants, cut into 4 mm × 4 mm-sized leaf discs on sterilized filter paper in laminar flow hoods (AIRTECH, Suzhou, China), and inoculated on a regenerative medium containing different concentrations of hormones. Each medium was used to inoculate 25 leaf discs, and the process was replicated 3 times. After 30 days, the number of indefinite buds with lengths greater than 2 mm was counted, and the budding leaf disc rate and the budding rate were calculated.

### 3.3. Assessment of Antibiotic Sensitivity of the Untransformed Plants

In this experiment, the concentrations and types of antibiotics used for the selection of Gojo-0 leaf explants were tested to determine the lethal doses of different types and concentrations of antibiotics. The sterile Gojo-0 leaves were cut into 4 mm × 4 mm pieces and inoculated into bud regeneration medium M1 with kanamycin concentrations of 0, 7.0, 8.0, 9.0, and 10.0 mg·L^−1^; hygromycin concentrations of 0, 2.0, 3.0, 4.0, and 5.0; and glufosinate-ammonium concentrations of 0, 0.3, 0.4, 0.5, and 0.6 mg·L^−1^. Twenty-five explants were inoculated per treatment, and each treatment was repeated 3 times. The selection pressure of antibiotics was calculated according to the percentage of browning and albino leaf disc budding rate after 30 days in the selected medium.

The shoots of sterile seedlings of Gojo-0 were inoculated into rooting medium (MS) containing kanamycin at concentrations of 0, 7.0, 8.0, 9.0, and 10.0 mg·L^−1^; hygromycin at concentrations of 0, 1.0, 1.5, 2.0, and 2.5 mg·L^−1^; and glufosinate-ammonium at concentrations of 0, 0.2, 0.3, 0.4, and 0.5 mg·L^−1^. Each treatment was used to inoculate 5 shoot explants and was repeated 3 times. After 15 days, the rooting status was examined to determine the concentrations of kanamycin, hygromycin and glufosinate-ammonium suitable for screening.

### 3.4. Agrobacterium-Mediated Transformation

Establishing a good selective system is critical for generating transgenic plants. In this study, the leaves of young Gojo-0 sterile seedlings aged approximately 40 days were cut into 300 leaf discs (4 mm × 4 mm) on sterilized filter paper in laminar flow hoods and precultivated on MS medium, which is most suitable for adventitious bud regeneration, for 3 days. The light intensity was 40–200 μmol·m^−2^·s^−1^, the light length was 16 h per day, and the temperature was 23 °C.

*A. tumefaciens* EHA105 harboring the pG10 plasmid was activated on YEB solid-plate medium containing 50 μg·mL^−1^ rifampicin and 50 μg·mL^−1^ kanamycin. After 2–3 days of constant-temperature cultivation, the colonies grown on the plate were inoculated in 50 mL of YEB liquid medium containing 50 μg·mL^−1^ rifampicin and 50 μg·mL^−1^ kanamycin and placed on a rocker at 28 °C and 200 rpm for 48 h until the OD_600_ reached 0.4–0.6. Then, the *Agrobacterium* culture was centrifuged in a 50 mL sterile tube at 4000 rpm for 15 min, the supernatant was discarded, and 30 mL of liquid MS medium was added to resuspend the *Agrobacterium* cells. The leaf discs were precultured for 3 days on solid medium and soaked in the infection solution for 8–10 min. During this period, the petri dish was gently shaken to ensure that the leaf discs were in full contact with the infection liquid. After infection, the leaf discs were removed, and excess infectant was aspirated with sterile filter paper. The leaf discs were inoculated on M1 adventitious bud regeneration medium and cultured at 23 °C for 3 days in a dark environment [37,38]. Then, the cocultured leaf discs were transferred to M1 solid medium containing 500 mg·L^−1^ carbenicillin for 7 days to eliminate excess *Agrobacterium* and inoculated into differentiation selection medium (M1 + 10 mg·L^−1^ kanamycin + 400 mg·L^−1^ carbenicillin). The cells were cultured for 3–4 generations, and the medium was changed every two weeks. Each time the solid medium was changed, the carbenicillin and kanamycin concentrations for screening were gradually decreased by 100 mg·L^−1^ and 2 mg·L^−1^, respectively.

After four screenings, the kanamycin-resistant buds were cultured on MS solid medium containing 12 mg·L^−1^ kanamycin and 150 mg·L^−1^ carbenicillin for rooting screening. Approximately 15 days later, the resistant buds began to take root, and the leaves of the rooting seedlings were selected for molecular detection.

### 3.5. PCR Detection

Leaf genomic DNA was isolated from genetically transformed plants and wild-type (WT) plants using a DNA Extraction Kit (Huayueyang, Beijing, China), followed by PCR analysis using specific primers (Table 4). The total DNA of the resistant plants was used as the template, plasmid DNA was used as the positive control, and the DNA of WT plants was used as the negative control. In all cases, PCR amplification was carried out in a 25 µL reaction mixture containing 100 ng of genomic DNA, 1.0 µL of each primer (10 µM), 2.5 µL of 10× PCR buffer, 2.0 µL of dNTP mix (2.5 mM), 0.2 µL of *rTaq* enzyme and 17.3 µL of ddH_2_O. The standard thermal cycling conditions were as follows: 94 °C for 3 min; 35 cycles of 94 °C for 10 s, 55 °C for 20 s, and 72 °C for 30 s; and finally 72 °C for 7 min. The PCR instrument was produced by Bio-Rad, model PTC-200. After the PCR amplification procedure, the product was analyzed by 1% (*w*/*v*) agarose gel electrophoresis.

### 3.6. GUS Staining

The transgene-positive seedlings were detected by a GUS staining kit (Huayueyang, Beijing, China), and the leaves of uninfected plants were taken as a negative control. The staining solution completely covered the leaves, and the cells were stained for 24 h at 37 °C in the dark. After staining, the GUS staining solution was discarded, and the samples were washed with 70%, 85% and 100% ethanol successively until the pigment completely faded and the leaves became white. The decolorized leaves were dried with filter paper and observed under a stereomicroscope.

### 3.7. Identification of the T-DNA Insertion Locus Using High-Throughput Sequencing

To confirm the insertion of T-DNA, we applied a high-throughput sequencing strategy to identify the T-DNA insertion locus. The detailed method is described below. First, the genomic DNA of transgenic line 2 was isolated using a DNA extraction kit. Then, 500 ng of gDNA was sent to a commercial service for Illumina sequencing (Yuanbaobio Biotech, Nanjing, China). After quality control using the customized script, the clean reads were aligned to the T-DNA sequence of the pG10 vector using the BWA-MEM algorithm [39]. The aligned reads were de novo assembled into contigs using SPAdes 3.12.0 [40]. The longest contig was compared with the T-DNA sequence, and the flanking sequence of the contig was considered the genomic sequence for insertion. Finally, the sequence was blasted against the reference genome of *C. seticuspe* (http://mum-garden.kazusa.or.jp/blast.html, accessed on 4 April 2022) to identify the T-DNA insertion locus.

### 3.8. Data Analysis

The data were sorted by Excel 2019, and the data were processed and analyzed by SPSS 26.0 software. The significance of differences across the treatment groups was evaluated by one-way ANOVA, and the least significant difference method (LSD) was used for multiple comparisons (*p <* 0.05).

## 4. Conclusions

In this study, the NAA and 6-BA concentrations in the regeneration system were 1.0 mg·L^−1^ and 0.2 mg·L^−1^, respectively, and could most effectively stimulate callus and shoot formation of leaf explants on regular MS medium. The best concentrations of kanamycin, hygromycin and glufosinate-ammonium were 10 mg·L^−1^, 2.5 mg·L^−1^ and 0.6 mg·L^−1^ for bud generation and 12 mg L^−1^, 1.5 mg·L^−1^ and 0.5 mg·L^−1^ for rooting. Clear amplified bands and obvious staining in transgenic plants could be observed by PCR detection and GUS staining, respectively, and the results of high-throughput sequencing further verified the feasibility of the regeneration and genetic transformation system.

In conclusion, Gojo-0 is expected to become a model plant for chrysanthemum due to its self-compatibility and pure diploid genome characteristics. In this study, Gojo-0 was used as the research object, and its regeneration and genetic transformation system was optimized to promote the development of chrysanthemum gene editing and germplasm resource innovation.

## Figures and Tables

**Table 1:** Effects of different types and concentrations of antibiotics on leaf disc explant differentiation of Gojo-0.

Types of Antibiotics	Concentration of Antibiotic (mg·L^−1^)	Callus Induction Rate (%)	Budding Leaf Disc Rate (%)	Budding Rate (%)
Kanamycin	0	100 ± 0 ^a^	77.33 ± 5.81 ^b^	114.67 ± 8.11 ^a^
	4.0	96.00 ± 2.31 ^ab^	22.67 ± 7.06 ^c^	36.00 ± 6.11 ^b^
	6.0	60.00 ± 2.31 ^d^	13.33 ± 7.06 ^cd^	16.00 ± 9.24 ^de^
	8.0	25.33 ± 3.53 ^f^	4.00 ± 4.00 ^de^	5.33 ± 5.33 ^de^
	10.0	8.00 ± 2.31 ^gh^	0 ± 0	0 ± 0
Hygromycin	0	100 ± 0 ^a^	78.67 ± 3.53 ^ab^	128.00 ± 8.33 ^a^
	1.0	87.67 ± 2.91 ^b^	9.43 ± 4.28 ^de^	12.62 ± 5.91 ^c^
	1.5	72.00 ± 5.29 ^c^	7.35 ± 2.15 ^de^	9.87 ± 3.34 ^de^
	2.0	42.67 ± 8.11 ^e^	3.51 ± 2.15 ^de^	3.51 ± 2.15 ^de^
	2.5	13.33 ± 2.40 ^g^	0 ± 0	0 ± 0
Glufosinate-ammonium	0	100 ± 0 ^a^	89.33 ± 3.53 ^a^	118.67 ± 7.06 ^a^
	0.3	85.33 ± 5.33 ^b^	24.00 ± 2.31 ^c^	33.33 ± 4.81 ^bc^
	0.4	54.67 ± 5.81 ^d^	13.33 ± 3.53 ^cd^	18.67 ± 4.81 ^cd^
	0.5	28.00 ± 4.62 ^f^	5.33 ± 3.53 ^de^	5.33 ± 3.53 ^de^
	0.6	0 ± 0	0 ± 0	0 ± 0

Budding leaf disc rate = the number of explants for adventitious bud regeneration/total number of inoculated explants × 100. Budding rate = the number of adventitious buds regenerated/leaf number of explants regenerated × 100. Callus induction rate = leaf disc number of differentiated callus/total number of inoculated explants. Different letters indicate expression levels that were significantly different at *p* < 0.05.

**Table 2:** Effects of different types and concentrations of antibiotics on leaf disc explant rooting of Gojo-0.

Types of Antibiotics	Concentration of Antibiotic (mg·L^−1^)	Frequency of Rooting (%)
Kanamycin	0.00	100 ± 0 ^a^
	6.00	33.3 ± 6.67 ^c^
	8.00	26.7 ± 6.67 ^c^
	10.00	14.4 ± 6.67 ^c^
	12.00	0 ± 0
Hygromycin	0.00	100 ± 0 ^a^
	1.50	13.3 ± 6.67 ^c^
	2.00	0 ± 0
	2.50	0 ± 0
	3.00	0 ± 0
Glufosinate-ammonium	0.00	100 ± 0 ^a^
	0.20	80 ± 11.55 ^b^
	0.30	60 ± 0 ^b^
	0.40	33.3 ± 6.67 ^c^
	0.50	0 ± 0

Frequency of rooting = the number of shoots rooting/total number of shoots × 100%. Different letters indicate expression levels that were significantly different at *p* < 0.05.

**Table 3:** Effects of different hormone proportions on the regeneration of Gojo-0.

Code	6-BA (mg·L^−1^)	NAA (mg·L^−1^)	Budding Leaf Disc Rate (%)	Budding Rate (%)
M1	1	0.2	96.00 ± 2.31 ^a^	162.67 ± 16.38 ^a^
M2	1	0.5	70.67 ± 9.33 ^bc^	92.00 ± 10.58 ^bc^
M3	1	0.8	13.33 ± 3.53 ^f^	16.00 ± 4.00 ^e^
M4	2	0.2	61.33 ± 8.74 ^bcd^	97.33 ± 20.95 ^bc^
M5	2	0.5	78.67 ± 8.74 ^ab^	124.00 ± 6.93 ^b^
M6	2	0.8	52.00 ± 2.31 ^cd^	90.67 ± 4.81 ^bc^
M7	3	0.2	14.67 ± 1.33 ^f^	16.00 ± 0 ^e^
M8	3	0.5	29.33 ± 9.61 ^ef^	41.33 ± 8.11 ^de^
M9	3	0.8	42.67 ± 9.33 ^de^	61.33 ± 15.03 ^cd^

Budding leaf disc rate = the number of explants for adventitious bud regeneration/total number of inoculated explants × 100%. Budding rate = the number of adventitious buds regenerated/leaf number of explants regenerated × 100%. Different letters indicate expression levels that were significantly different at *p* < 0.05.

**Table 4:** Primer sequence for identification.

Primer Name	Sequence (5′–3′)	Amplicon Length	Application
GUS/NPTII-F	GGGCAACAAGCCGAAAGA	252 bp	Evaluate transgenic plants of Gojo-0
GUS/NPTII-R	TGAGCGTCGCAGAACATTACAT		
pG10-Backbone-F	GCCTTCTTGACGAGTTCTTCTGA	505 bp	Evaluate pG10-RUBY vector backbone
pG10-Backbone-R	CATGCTACCCTCCGCGAGA		
Cse2.0_LG3-F	AGGGTGGTGGTCATTTAGCCT	~700 bp	Evaluate insertion site of line 2
pG10-LB-R	TGGGCTGACCGCTTCCTC		

## Data Availability

The sequencing data presented in this study have been submitted to the NCBI Sequence Read Archieve Centre (SRA) (https://www.ncbi.nlm.nih.gov/sra, accessed on 4 April 2022) under Bioproject PRJNA826694.

## Supplementary Materials

The following supporting information can be downloaded at: https://www.mdpi.com/article/10.3390/ijms231911426/s1.
